# Experimental evidence of shear waves in fractional viscoelastic rheological models

**DOI:** 10.1038/s41598-022-11490-4

**Published:** 2022-05-06

**Authors:** Antonio Gomez, Antonio Callejas, Guillermo Rus, Nader Saffari

**Affiliations:** 1 Mechanical Engineering Department, University College London, London, WC1E 7JE UK; 2 ibs.GRANADA, Instituto de Investigación Biosanitaria, 18012 Granada, Spain; 3 Structural Mechanics Department, University of Granada, 18071 Granada, Spain; 4 Excellence Research Unit “Modeling Nature” (MNat), University of Granada, 18071 Granada, Spain

**Keywords:** Biomedical engineering, Mechanical engineering

## Abstract

Fractional viscoelastic rheological models, such as the Kelvin Voigt Fractional Derivative model, have been proposed in the literature for modelling shear wave propagation in soft tissue. In this article, our previously developed wave propagation model for transluminal propagation based on a Kelvin Voigt Fractional Derivative wave equation is experimentally validated. The transluminal procedure uses the transmission and detection of shear waves through the luminal wall. The model was compared against high-speed camera observations in translucent elastography phantoms with similar viscoelastic properties to prostate tissue. An ad hoc cross-correlation procedure was used to reconstruct the angular displacement from the high-speed camera observations. Rheometry and shear wave elastography were used for characterising the shear wave velocity dispersion curve for the phantoms. Fractional viscoelastic properties were derived after fitting the dispersion curve to its analytical expression. Propagation features and amplitude spectra from simulations and high-speed camera observations were compared. The obtained results indicate that the model replicates the experimental observations with acceptable accuracy. The model presented here provides a useful tool to model transluminal procedures based on wave propagation and its interaction with the mechanical properties of the tissue outside the lumen.

## Introduction

**Figure 1:**
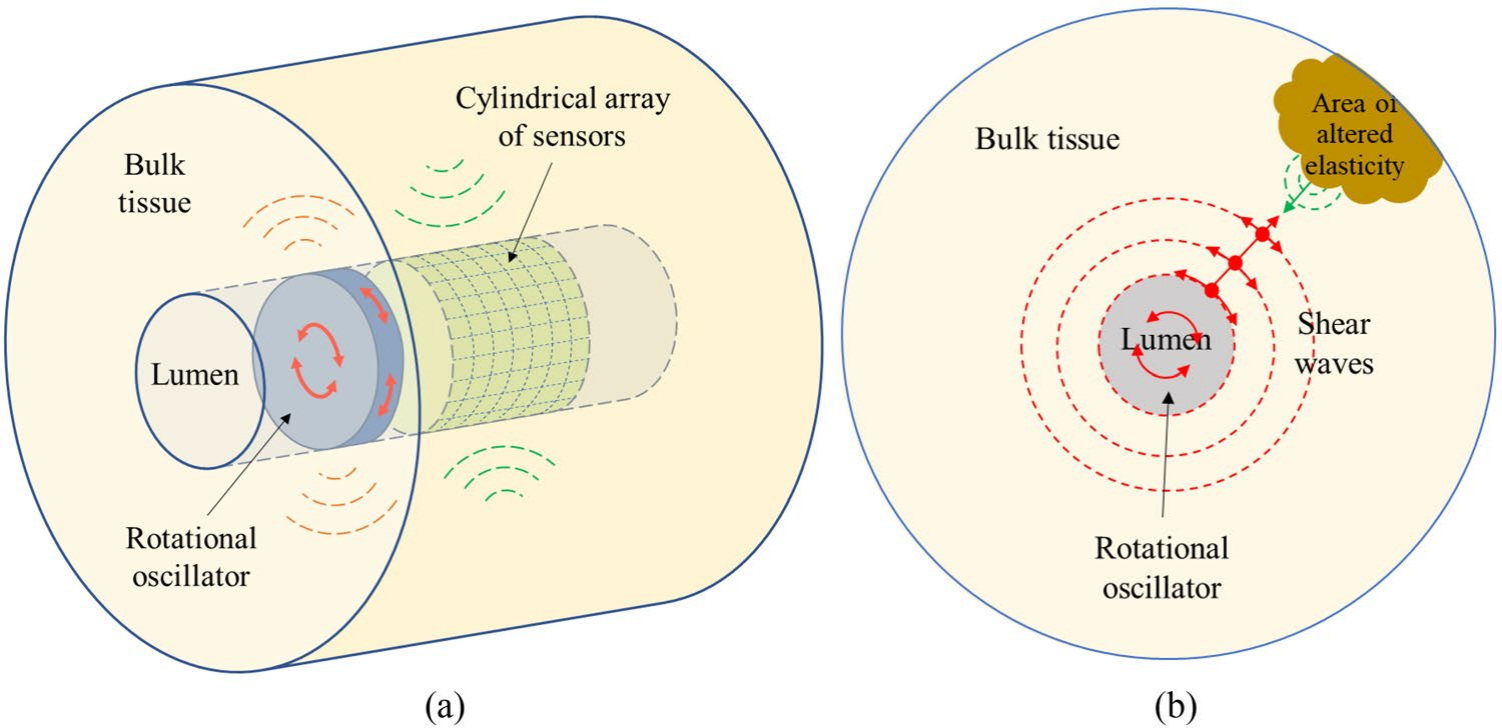
(**a**) Conceptual idealisation of the transluminal elastography approach. The transluminal probe, composed for at least one rotational oscillator and a cylindrical array of sensors, is inserted through the lumen. (**b**) Cross section schematic view of the transluminal approach. Shear waves propagates radially from the rotational oscillator disk emitter. Particles vibrate in an arc-shaped manner perpendicular to the propagation direction. Echoes are generated as the shear waves interact with the area of altered viscoelastic properties. *Source*: Gomez et al.^4^.

Elastography refers to a set of imaging techniques that evaluates the elasticity of tissue for medical purposes^1,2^. Many clinical applications of elastography are performed extracorporeally using surface ultrasound transducers, however, there are cases where the target tissue could be better accessed from a body lumen^3^. A new procedure for performing transluminal elastography was presented in a previous article^4^. The technique is based on the transmission of shear waves into the bulk of the tissue by applying oscillatory rotational forces on the luminal wall (see Fig. 1). This way of transmission generates a pseudo-spherical propagation pattern of shear waves, with arc-shaped particle vibration, that interacts with regions of altered viscoelasticity of the target tissue. This interaction partially leads to reflected energy that travels back to the lumen wall where it can be detected^3^.

A wave propagation model was presented in a previous article as the first step in the development of the new transluminal procedure^4^. The model was based on a Kelvin Voigt Fractional Derivative (KVFD) constitutive law^5,6^ (see Eq. 1), and was solved by a Finite Difference Time Domain (FDTD) scheme. An in silico example based on prostate cancer detection was used to test the wave propagation model and illustrate the new transluminal approach, however, the wave propagation model was not validated. According to the KVFD constitutive law, the stress $\sigma$ depends on the fractional time derivative of order $\alpha$ of the strain $\varepsilon$, as shown in the following equation for the shear stress case:

1 $$\begin{aligned} \sigma = \mu \varepsilon + \eta _s \frac{\partial ^\alpha \varepsilon }{\partial t^\alpha } \end{aligned}$$

where $\mu$ is the second Lamé’s parameter and $\eta _s$ is the shear viscosity. $\mu$ is also known as the shear modulus. Models based on fractional linear viscoelastic constitutive laws such as the KVFD can reproduce the power law behaviour of cumulative multiple relaxation processes observed in longitudinal and shear wave propagation in soft tissue^5,6^. Furthermore, fractional viscoelastic models can also reproduce absorption power laws with the exponent taking values from a continuous range from 0 to 2^7–9^.

Tissue-mimicking phantoms are commonly used in medical imaging research prior to testing actual tissue. In the field of elastography, early phantom studies analysed the elastic properties of commercial tissue-like materials available for ultrasound testing. However, their elastic properties differed from those of soft tissues, thus causing the investigation of phantoms specifically designed for elastography^10,11^. A vast variety of phantom recipes have been developed during the last three decades^12^. Elastography phantoms are commonly made from a water base such as gelatine, agar or Polyvinyl Alcohol (PVA); an oil base, such as paraffin-gel; or a mixture, an oil-in-hydrogel. Apart from the base components, additives can be added to provide the phantom with other specific features, for example to increase backscattering or to control the proliferation of fungi and bacteria^11^. Elastography prostate phantoms can be found in the literature. For example, gelatine-based phantoms to test a vibro-elastography technique^13^, or phantoms with stiff inclusions made of higher concentration of gelatine to test detection by magnetic resonance elastography^14^. Other prostate phantoms using different materials can also be found. For instance, using agar for the normal prostate background and polyacrylamide to mimic stiff inclusions^15^, a tissue-equivalent ultrasound prostate phantom (053-MM, CIRS Inc., Norfolk, VA, USA)^16^ and using PVA at different concentration and freezing-thawing cycles to create stiff inclusions^3^.

Validation of a wave propagation model can be accomplished by comparing model simulations against experimentally measured features of the wave propagation. Optical techniques using lasers have been used to measure shear waves generated by Acoustic Radiation Force (ARF) in elastography phantoms. In one of the first reported experimental observations of shear waves generated by ARF, a laser beam was used to successfully observe the shear waves and measure their group velocity^17^. The laser was partially focused on the edge of an embedded particle in a phantom, so that the amount of light passing through was linked to the magnitude of the particle vibration. Laser vibrometry has been used to measure the velocity dispersion curve of the shear waves generated by oscillatory ARF^18^. The laser measured the vibration of micro mirror particles embedded in the phantom, thus allowing the measurement of the phase shift of narrow band shear waves between two different locations. Optical tracking by High-Speed Camera (HSC) has also been used for validation purposes, for which a minimum level of transparency is a required phantom feature. An example is the investigation of the mechanical response to ARF-based elastography excitation in a gelatine-based phantom^19^. The optical system consisted of a microscope and an attached high-speed camera. Shear waves were successfully observed by tracking the displacement of an embedded single particle. Later, another similar optical tracking system was used to experimentally confirm displacement underestimation of US systems when using ARF-based elastography^20^. A third similar HSC tracking system was used to characterise the amplitude of the displacements and the phase shift of shear waves induced by ARF^21^.

The various examples shown above demonstrate that optical techniques are suitable to capture the vibrations of single particles. However, observing the mechanical response of a cloud of particles would increase the amount of information and, hence, would reduce the error in the reconstruction of the particle vibration features. This ability is particularly required in the case of observing shear wave transmitted according to the transluminal procedure, where particle vibration is not straight, as it follows an arc-shaped pattern with the lumen axis as the centre of rotation. A HSC-based technique was chosen due to its simplicity of use and its ability to simultaneously observe a cloud of particles. A gelatine-based solution from a modified combination of recipes^14,22^ was chosen to mimic the prostatic tissue while keeping a reasonable level of transparency.

This article contributes an HSC approach for measuring the vibration of a cloud of particles due to shear wave propagation in translucent phantoms, for which, the process of fabrication is meticulously detailed. The article describes the experimental validation of the KVFD-based wave propagation model developed for a transluminal elastography procedure presented in our previous work^4^. The validation of the model was achieved by comparing model simulations against HSC observations of shear waves propagated in translucent gelatine-based phantoms containing stiff inclusions. Following the prostate cancer example used for illustrating the wave propagation model in the previous article^4^, prostatic normal and cancerous tissue viscoelastic properties were used to fabricate the phantoms and validate the wave propagation model.

## Materials and methods

### Wave propagation model

**Figure 2:**
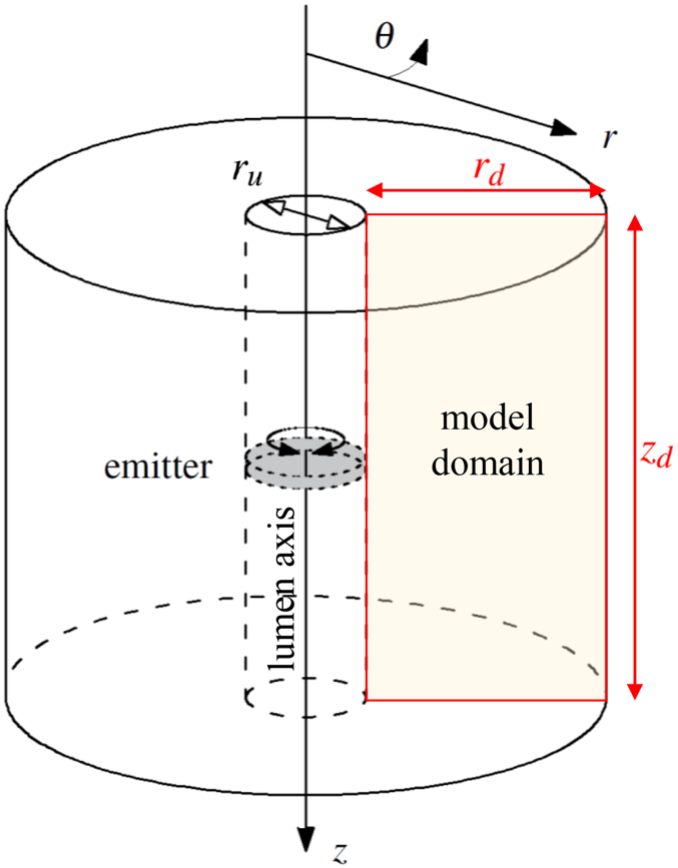
Geometry and system of coordinates used in the wave propagation model. The grey disk represents a rotational emitter placed within the lumen. Spatial domain of the axisymmetric model contoured in red. $r_d$, $z_d$ and $r_u$ are defined in Table 1. *Source*: Gomez et al.^4^.

The wave propagation model uses cylindrical coordinates and considers axial symmetry with respect to the lumen (see Fig. 2). Conservation of momentum^23^ (Eq. 2), linear strain-displacement relationships^24^ (Eqs. 3 and 4) and the KVFD constitutive law^25^ (Eqs. 5 and 6) build up the system of equations that are solved using a FDTD scheme. The reader is referred to Gomez et al.^4^ for full detail of the model.

2 $$\begin{aligned}&\rho \frac{\partial ^2 u_\theta }{\partial t^2} = \frac{\partial \sigma _{r\theta }}{\partial r} + \frac{\partial \sigma _{\theta z}}{\partial z} + \frac{2}{r} \sigma _{r\theta } \end{aligned}$$

3 $$\begin{aligned}&\varepsilon _{r\theta } = \frac{1}{2}\left( \frac{\partial u_\theta }{\partial r} - \frac{u_\theta }{r} \right) \end{aligned}$$

4 $$\begin{aligned}&\varepsilon _{\theta z} = \frac{1}{2} \frac{\partial u_\theta }{\partial z} \end{aligned}$$

5 $$\begin{aligned}&\sigma _{r \theta } = 2 \left( \mu + \eta _s \frac{\partial ^\alpha }{\partial t^\alpha } \right) \varepsilon _{r \theta } \end{aligned}$$

6 $$\begin{aligned}&\sigma _{\theta z} = 2 \left( \mu + \eta _s \frac{\partial ^\alpha }{\partial t^\alpha } \right) \varepsilon _{\theta z} \end{aligned}$$

where $\rho$ is the local density of the medium and *u* is the vector of displacements. The subscripts $r,z,\theta$ of $\sigma$, $\varepsilon$ and *u* represents the coordinate components. The boundary conditions were: the excitation source at the points on the luminal wall where the rotational oscillator disk is placed, and the absence of shear stress on the rest of the luminal wall with the remaining domain contour as free boundaries. All the relevant model parameters are listed in Table 1 following the nomenclature used in Gomez et al.^4^.

**Table 1:** Values of the model parameters.

Model parameter	Description	Value
**Domain size**		
$r_d$	Radial dimension of the domain	25.00 mm
$z_d$	Depth dimension of the domain	60.00 mm
$r_u$	Radius of the lumen	5.00 mm
**Discretisation parameters**		
$\Delta r$	*r* spatial dimension interval	150.00 $\upmu {\text {m}}$
$\Delta z$	*z* spatial dimension interval	150.00 $\upmu {\text {m}}$
$\Delta t$	Time interval	20.00 µs
$t_T$	Total time of simulation	25.00 ms
$t_L$	Time reference for *L* param.	1.00 ms
**Emitter setup**		
$n_e$	Number of emitters	1
$z_e$	*z* coordinate of the emitter	15.00 mm
$l_e$	Length of the emitter	4.00 mm
**Stiff inclusion features**		
$r_c$	*r* coordinate of the inclusion centre	20.00 mm
$z_c$	*z* coordinate of the inclusion centre	15.00 mm
$_c$	Diameter of the inclusion	10.00 mm

Further detail in Gomez et al.^4^.

### Phantom fabrication

The use of a HSC-based technique for measuring the vibration of a cloud of particles within a phantom imposes the requirement of a minimum level of transparency, which is achieved by using gelatine. Gelatine-based phantoms were fabricated from a combination of several recipes from the literature^14,19,20,22,26^. Details of the components used and their concentration per 100 mL of distilled water are shown in Table 2. Bovine gelatine was the gelation component. Formalin was added as a cross-linking agent, which elevates the melting point of the gelatine (originally around 32 $^\circ$C) thus stabilising the mechanical properties of the phantom to minor room temperature variations. Potassium sorbate (K-sorbate) was included to preserve the phantom from early decay due to bacterial and fungal activity. Additives for tuning viscosity and acoustic properties were avoided after observing a drastic drop in the transparency of the phantom when added. Four different batches of the gelatine solution were produced. The batch for the phantom background had a 9% wt/wt concentration of gelatine (weight relative to that of the final solution, i.e., including all the phantom components). The other three batches were prepared for the inclusions that would mimic prostatic tumours. Concentrations of the components for each batch are summarised in Table 3.

**Table 2:** Components and concentration used for the background of the phantom per 100 mL of distilled water.

Component	Quantity	Supplier, type
Gelatine	10 g	Sigma Aldrich, Bovine skin gelatine 225 bloom
Formalin	0.24 mL	Sigma Aldrich, Formaldehyde sol. 37% weight (wt) in H$_2$O
K-sorbate	1.62 g	Alfa Aesar, Potassium sorbate, 99%
H$_2$O	100 mL	Laboratory distilled water

**Table 3:** Gelatine concentration and amount of each component for different phantom batches.

Batches	Gelatine (wt/wt) (%)	Amount per 100 mL of water		
		Gelatine (g)	Formalin (mL)	K-sorbate (g)
Background	9	10	0.24	1.62
Inclusion 1	12	14	0.24	1.62
Inclusion 2	14	17	0.24	1.62
Inclusion 3	16	20	0.24	1.62

**Figure 3:**
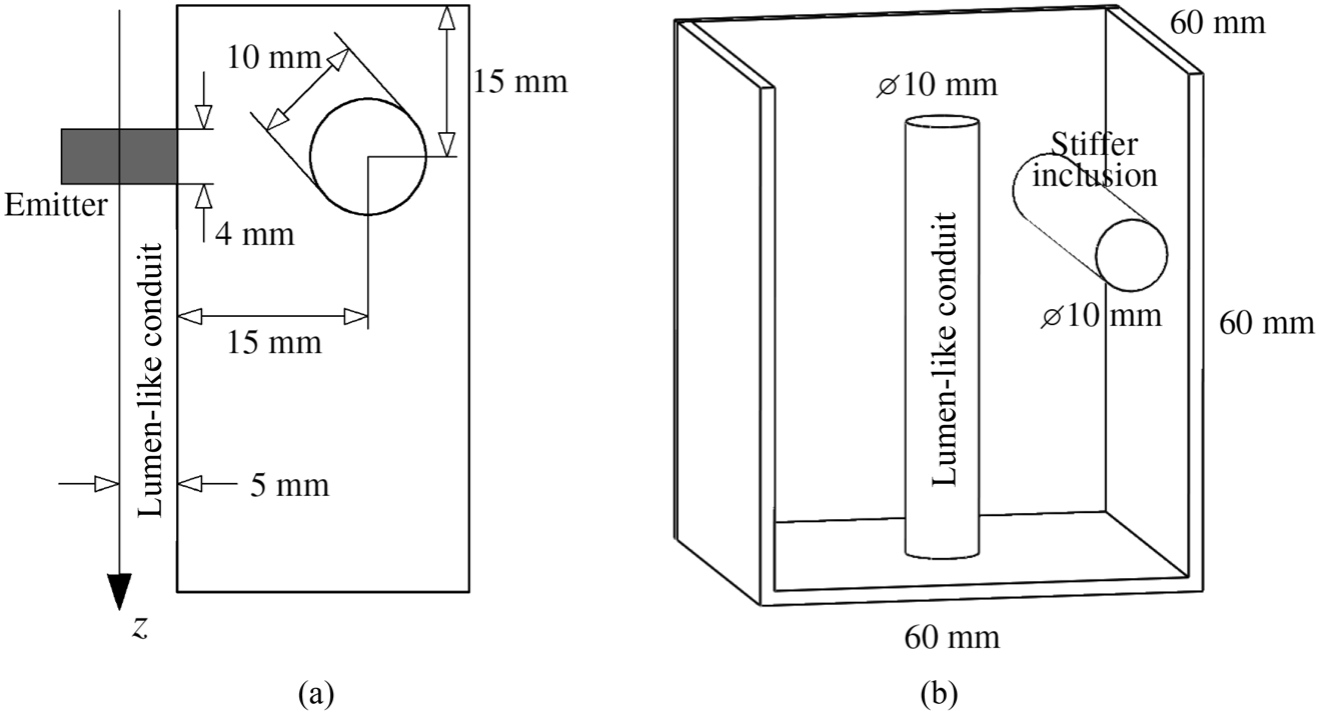
(**a**) Configuration and dimensions of the wave propagation model scenario used for designing the phantoms. (**b**) Mould design for casting the phantoms. The front face has been removed to ease visualisation.

Cuboids moulds of 60 $\times$ 60 $\times$ 60 mm for casting the phantoms were 3D printed using Polylactic Acid (PLA) as separate assembling parts, which facilitated the extraction of the phantom after gelation. Two solid straight cylindrical rods were added to the design (Fig. 3b). One rod was vertically centred intended to leave a conduit for the lumen-like passage. The other, perpendicular and separated 10 mm from the vertical one, was designed to leave an empty space to be filled later with a higher gelatine concentration solution to form the inclusion. Acetate film sheets (HP Premium InkJet C2834A, Hewlett-Packard, CA, USA) were stuck onto the interior side of each face of the cuboid moulds to ensure perfect planar ends, thus avoiding optical aberrations. The dimensions of the 2D scenario are shown in Fig. 3a. The diameter of the lumen-like conduit was 10 mm, in order to allow the use of an emitter prototype fabricated by University of Granada (Spain) on another collaborative project. The diameter of the emitter was 11 mm. The difference of 1 mm ensured good mechanical contact between emitter and phantom surface. The diameter of the inclusion was also set as 10 mm, a value within the order of the size of prostate tumours^27^.

**Figure 4:**
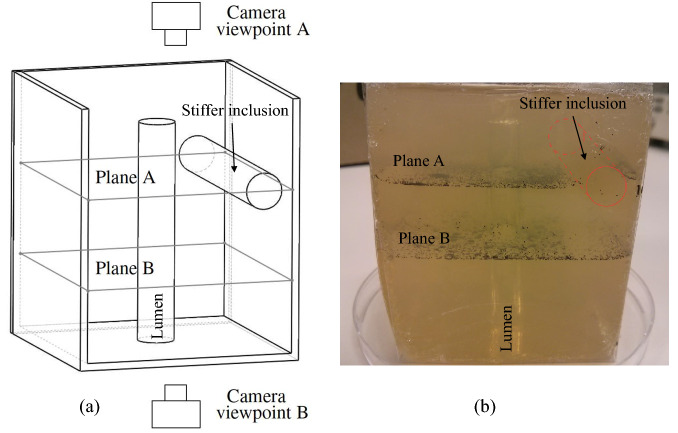
Approximate location of the planes with patterns of particles in the (**a**) the mould design for the phantom. Plane A contained a section of the inclusion whilst plane B did not. The viewpoints for the camera differed for each plane with particles. (**b**) Completed phantom placed on a Petri dish. Both planes of particles are visible, as well as the vertical lumen conduit and the stiffer inclusion (dashed red cylinder).

Planar patterns of visible particles of size a fraction of the expected wavelength were embedded in the phantom. The visible particles consisted of basalt microspheres (Whitehouse Scientific Ltd., Chester, UK) of sizes between 180 and 212 $\upmu {\text {m}}$. Particles were randomly distributed and contained in planes perpendicular to the lumen axis, where the arc-shape particle vibration was better observable (Fig. 1b). The two planes were set at two different *z* coordinates, as indicated in Fig. 4: one containing the stiff inclusion, labelled as plane A, and another without inclusion labelled as plane B.

Three phantoms were fabricated, each one containing an inclusion of different gelatine concentration as indicated in Table 3. The background of all the phantoms was made from the same gelatine batch. The gelatine batch was prepared as follows (quantities are shown in Table 2): (1) K-sorbate was added to the distilled water while stirring; (2) the mixture was heated up at a rate of approximately 1 $^\circ$C per minute; (3) gelatine powder was gradually added to the mixture until it was fully dissolved; (4) the mixture was covered and heated up to 85 $^\circ$C at a rate of approximately 1 $^\circ$C per minute, after which, the temperature was maintain between 85 and 90 $^\circ$C for 90 min; (5) the mixture was cooled to 40 $^\circ$C at a rate of approximately 1 $^\circ$C per minute; (6) formalin was added to the mixture while stirring gently. Part of the solution was poured into the mould to reach the level of plane B (see Fig. 4). The rest was kept at 40 $^\circ$C. The basalt particles were carefully sprinkled over the mixture after gelation of the surface. The temperature of the mixture in the mould was between 37 and 38 $^\circ$C). More mixture was carefully poured using a syringe onto the first section created to reach the level of plane A. The rest of the mixture was poured until totally filling up the mould. The filled mould was left to rest at room temperature (21 ± 1 $^\circ$C) for 12 h. After which, the phantom was carefully extracted from the mould. A new mixture with higher gelatine concentration was made. The new mixture was poured using a syringe into the empty cylindrical cavity left to form the stiff inclusion. The phantom was left to rest at room temperature for 30 min before being wrapped with plastic foil and stored in a sealed container at 4 $^\circ$C. Figure 4b shows one of the phantoms with stiff inclusion out of the mould. The inclusion did not contain basalt particles (see dashed red cylinder in Fig. 4b) because the patterns of particles were formed before filling up the cavity for the inclusion.

### Rheological characterisation of the tissue-mimicking materials

Viscoelastic properties of the different gelatine batches were characterised using a rheometer (MCR 300, Physica-Anton Paar, Graz, Austria). Small amounts of gelatine mixture from the same batches prepared for the phantoms were gelled into small Petri dishes. The same day of the HSC tests, disk-shaped samples of 20 mm diameter and 2-3 mm thick extracted from the Petri dishes were tested in the rheometer. The rheometry tests were carried out at a constant temperature of 20 $^\circ$C, the same temperature at which the HSC tests were carried out. Measurements for loss of moisture were not considered necessary due to the short duration of the whole procedure, approximately 5 min per sample. Preconditioning of the samples was performed as in^28,29^, by inducing five cycles of low amplitude, 1% of shear strain, at a low shear rate, 0.1%/s. Three samples were tested per each type of gelatine batch. Each sample was consecutively tested four times.

**Figure 5:**
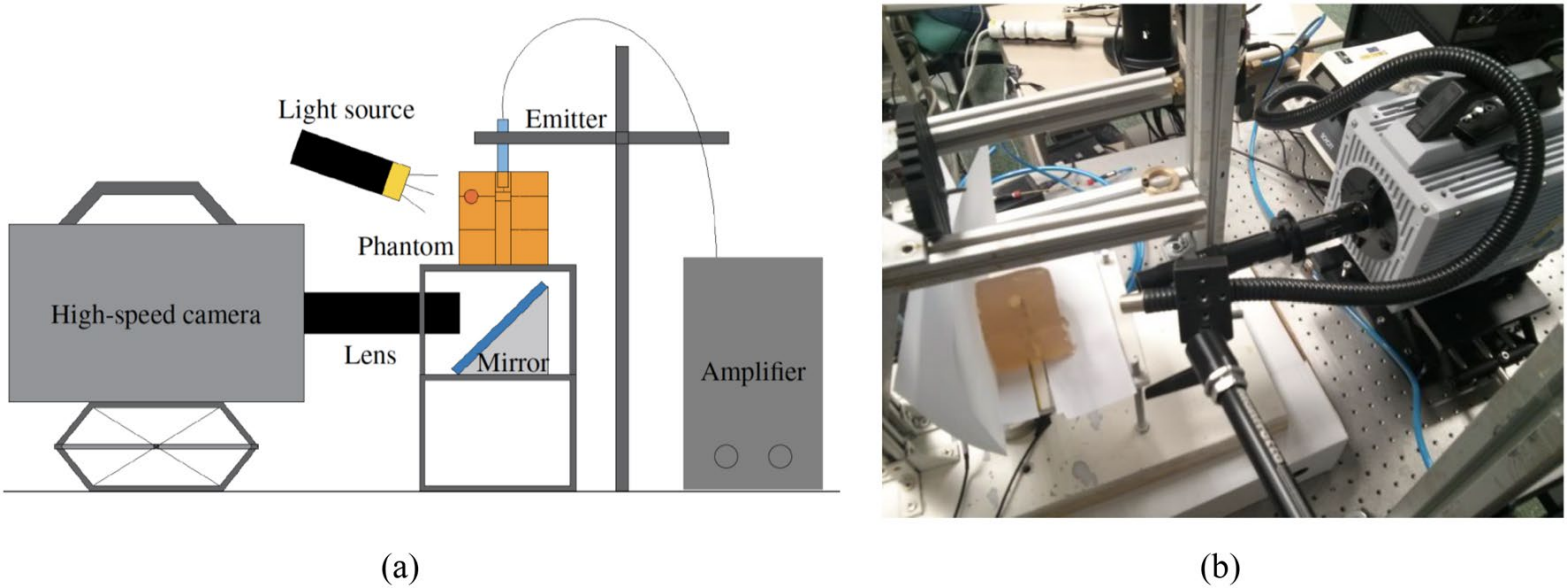
(**a**) Scheme of the setup for the HSC tests. The emitter (in blue) was held inside the phantom (in orange) through the lumen conduit. (**b**) Setup for the HSC tests. Phantom at the left side of the picture on top of the white platform. HSC and light source at the right side pointing towards the phantom.

A HSC testing approach was used to measure the displacement of the particles embedded in the two planes within the phantoms, due the propagation of shear waves generated by the transluminal procedure. The tests were carried out at the Fluid Mechanics Lab at University of Jaen (Jaen, Spain). The general setup is shown in Fig. 5. Each phantom was placed on an elevated platform with the lumen conduit vertically aligned. The platform presented a centered aperture to allow the visualisation of the phantom from the bottom side. A bespoke oscillatory rotational emitter was introduced into the lumen conduit and held in position by a clamping structure. The emitter was similar to that used in a previous experimental study^3^, consisting of a 11 mm diameter 3D printed PLA disk attached to the shaft of an electromagnetic rotational motor. The driving signal for the actuator was generated by a custom function generator, designed and fabricated by the Ultrasonics Lab at University of Granada (Granada, Spain)^30^. Dark spots were painted onto the disk emitter to allow the reconstruction of its rotation. A light source KL-2500-LCD (Schott North America Inc., NY, USA), guided by a flexible fibre optic bundle, was directed towards the phantom. A Fastcam SA1 high-speed camera (Photron Inc., San Diego, California, USA) with a zoom objective VZM™450i (Zoom Imaging Lens, Edmund Optics Inc., Barrington, USA) was positioned pointing towards a 45$^\circ$ mirror that was placed below the platform. A high-precision translation stage of 10 $\upmu {\text {m}}$ resolution was used for positioning the camera set. Fine focusing was achieved by manually adjusting the distance between the camera and the phantom while visually evaluating the sharpness of the planar pattern of particles on the monitor.

The driving signal for the actuator consisted of a train of 10 pulses, each one consisting of a single sinusoidal cycle. The separation time between pulses was 100 ms, which was considered sufficiently long to allow the propagating waves to vanish before the new transmission. The fundamental frequency of each pulse took incremental values from 100 to 1000 Hz. The maximum voltage amplitude of the excitation was set at 25 V. The signals were amplified using a low-frequency amplifier MA-25T (Fonestar Sistemas S.A., Barcelona, Spain). The function generator and the HSC were synchronised. An initial 5 V spike, sent before the excitation signal, triggered the camera. The camera filmed at a rate of 10,000 fps with a resolution of 720 $\times$ 720 pixels. Pixel size ranged between 16 and 18 $\upmu {\text {m}}$. The conversion value from pixel to spatial dimension was calculated for each recording by measuring features with known size on the front face of the disk emitter immediately before sending the driving signal.

Potential heating from the light source was kept under control. The light source was exclusively switched on for the filming time, i.e. 1 s. The light source used a fibre optic bundle to direct the light, which allowed to keep the main heat source at 80 cm from the phantom. In addition, the distance between the tip of the fibre optic bundle and the phantoms was about 20 cm. Temperature in the laboratory was maintained at 20 $^\circ$C. Temperature checks on the surface of the phantom were regularly performed between tests, observing variations of ± 0.3 $^\circ$C. It is reasonable to consider that the phantom experienced negligible temperature variation during this time, as the light source position and room conditions were carefully set. Furthermore, the addition of formalin to the mixture also improved the thermal stability of the phantom. Therefore, it could be assumed that the mechanical properties of the phantom were not affected during the tests.

### Particle vibration tracking method

The frames from the camera recordings were processed to improve image clarity using the open-source image processing software ImageJ (ImageJ, US National Institutes of Health, Maryland, USA)^31^. The reconstruction of the particle displacement was performed over the intersecting line between planes A and B and the plane of propagation of the 2D model. These lines are shown in Fig. 4a as dashed lines. The intersection lines are henceforth referenced as lines of analysis A and B, depending on the plane of particles from which they originated.

**Figure 6:**
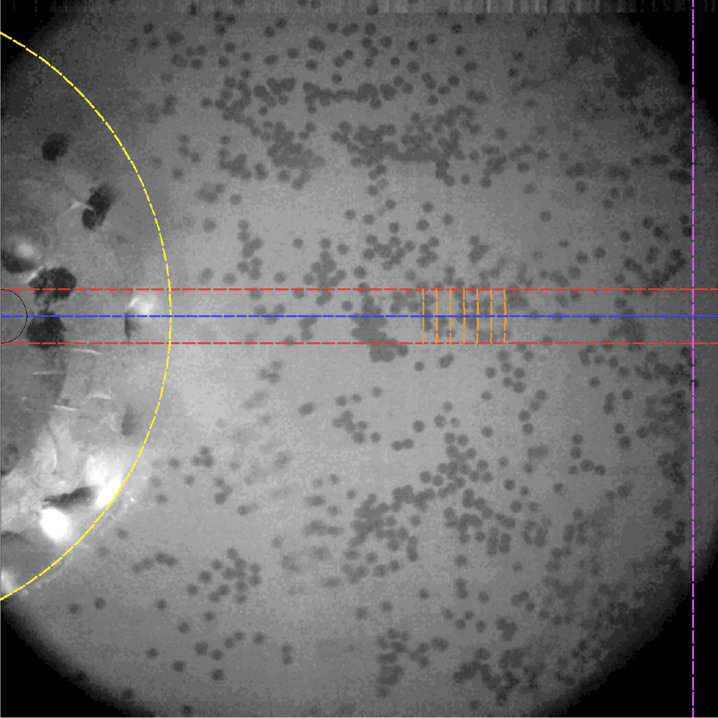
Schematic representation of the elements involved in the particle tracking method. The contour of the disk emitter and the stiff inclusion are coloured in yellow and purple, respectively. The area of interest for reconstructing the particle displacement due to the shear wave propagation falls between the two red dashed lines. The blue line represents the line of analysis. Examples of sections for calculating the cross-correlation are shown in orange.

A custom particle tracking algorithm, based on a cross-correlation technique, was developed for reconstructing the true displacement of particles due to the shear wave propagation from the camera recordings. The algorithm was developed using MATLAB^®^ (Release 2017a, MathWorks, Natick, United States). A reference frame was first selected, which corresponded to the resting state prior to the excitation. From the reference frame, the axis of rotation of the emitter was calculated as the centre of the disk. Then, the area of interest that contains the line of analysis was set (region between the two red dashed lines in Fig. 6, with 1.5 mm of separation between both lines). Frame by frame, small quasi-rectangular sectors 100–150 $\upmu {\text {m}}$ wide (contoured in orange colour in Fig. 6) were cross-correlated with rotated variants of the reference frame, until the entire area of analysis was covered (rotation with respect to the centre of the disk emitter). Then, the angular displacement of each sector was estimated by finding the angle that yielded the maximum coefficient of correlation. After this point, the procedure was repeated for the entire frame sequence from the recording. Finally, the angular displacement was transformed into linear displacement $u_\theta$, which provides a time-space representation of the particle vibration generated by the shear wave propagation along the lines of analysis.

### Validation approach

Shear KVFD viscoelastic parameters of the phantoms, obtained from the rheological characterisation and the HSC tests, were used to build up the equivalent in silico version in the 2D wave propagation model. The phase shear wave velocity $c_s$ was calculated from the HSC tests based on the following expression^32,33^:

7 $$\begin{aligned} c_s \left( \omega \right) = \frac{\omega \Delta r}{\Delta \phi \left( \omega \right) } \end{aligned}$$

where $\Delta \phi (\omega )$ is the phase change over the travelled distance $\Delta r$ as a function of the angular frequency $\omega$. The frequency dependent angle phase $\phi (\omega )$ was extracted from the Fast Fourier Transform (FFT) of the reconstructed particle displacement calculated at each spatial point of the line of analysis. The fraction $\Delta \phi / \Delta r$ was obtained as the gradient of a polynomial function of degree one fitted to the phase shift for each frequency component along the propagation distance.

In the case of the rheological characterisation, values of the shear wave velocity $c_s$ were calculated using the theoretical expression^34^:

8 $$\begin{aligned} c_s(\omega ) = \sqrt{\frac{2 \left( G'^2(\omega )+G''^2(\omega ) \right) }{\rho \left( G'(\omega ) + \sqrt{G'^2(\omega )+G''^2(\omega )}\right) }} \end{aligned}$$

where $\rho$ is the density, and $G'$ and $G''$, the real and imaginary parts of the complex shear modulus $G^*$, are the storage and loss shear moduli, respectively.

9 $$\begin{aligned} G' (\omega )= & {} \mu + \eta \omega ^\alpha \cos \left( \frac{\alpha \pi }{2} \right) \end{aligned}$$

10 $$\begin{aligned} G'' (\omega )= & {} \eta \omega ^\alpha \sin \left( \frac{\alpha \pi }{2} \right) \end{aligned}$$

The full velocity dispersion curve was built up by combining values derived from both the rheological and HSC tests^35^. The result was fitted by the theoretical KVFD expression for the shear wave velocity (Eqs. 8, 9 and 10^36^) to obtain the shear KVFD viscoelastic parameters. Details of this constitutive law are shown in the previous article describing the wave propagation model^4^. The Curve Fitting module of MATLAB^®^ was used.

The wave propagation simulations were set up by using the calculated shear KVFD parameters and the geometry shown in Figs. 3 and 4. Time and space discretisation was set as in the prostate cancer simulated scenario shown in the previous article^4^. The excitation signals used in the simulations corresponded to the $u_\theta$ displacement at the contact between disk emitter and lumen wall, reconstructed from each HSC test using the particle vibration tracking method. Boundary conditions around the domain were set as fully reflective to simulate the phantom-air interface. A correlation analysis based on the Pearson correlation coefficient $r_{Pearson}$ was used to compare the simulations and the HSC tests results in the time-space domain. A second validation approach based on the frequency-space domain, using the root-mean-square error (RMSE), was used to quantify the difference between amplitude spectra coming from the HSC tests and the simulations.

## Results

### Rheological tests results

**Figure 7:**
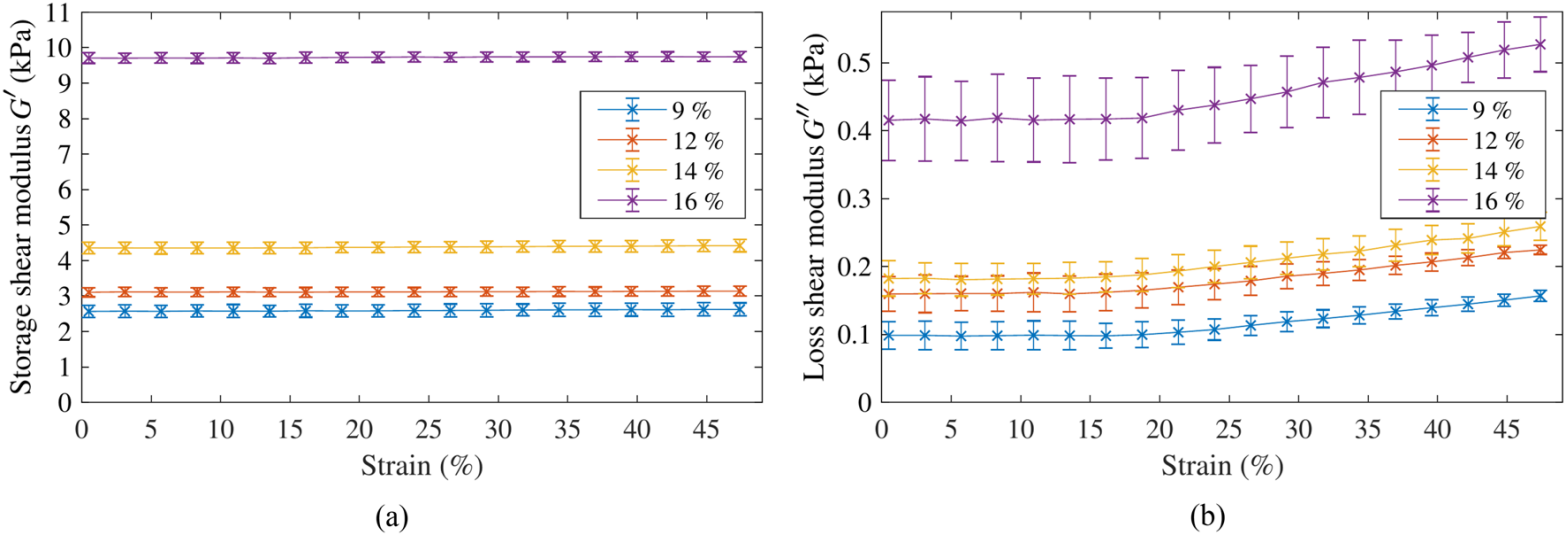
(**a**) Storage shear modulus and (**b**) loss shear modulus, as a function of the strain for the different gelatine concentration.

Rheometry tests were carried out on the four gelatine batches containing different gelatine concentration. Figure 7 shows the resulting storage $G'$ and loss $G''$ moduli from the strain sweep oscillatory tests. The samples were tested at 1, 2 and 4 Hz with maximum strain ranging from 0.5 to 49%. Figure 7 shows averaged results for all the samples with standard deviation bars using the three frequencies. The linear viscoelastic regime is identified as the range of strain where the complex shear modulus remains constant, independently of the maximum strain used during the test^29^. The storage modulus was practically constant for the whole range of strain tested (see Fig. 7a). However, the loss modulus started to become dependent on the strain at around a strain of 15%. Therefore, strain values below 15% were considered to be within the linear viscoelastic regime.

**Figure 8:**
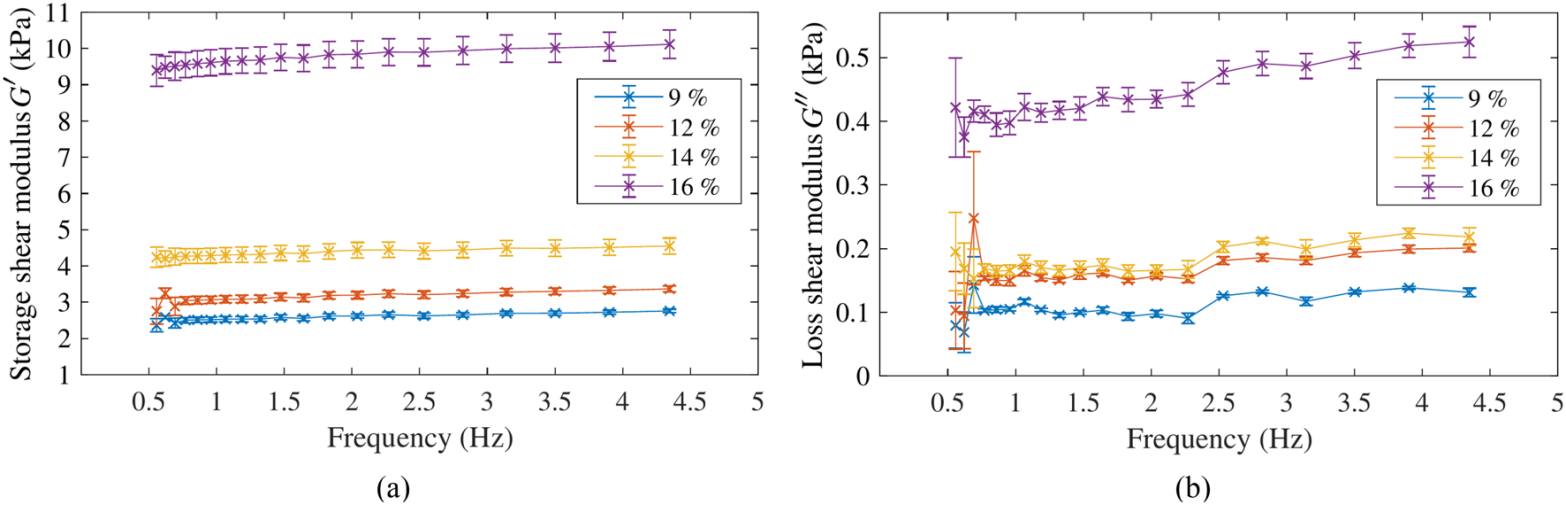
(**a**) Storage shear modulus and (**b**) loss shear modulus, as a function of frequency for the different gelatine concentration.

The frequency sweep tests characterised $G'$ and $G''$ as functions of frequency. These tests were performed for each gelatine batch, using a maximum strain of 1%, therefore within the linear viscoelastic regime, and for frequencies ranging from 0.5 to 50 Hz. Figure 8 shows the results in terms of mean and standard deviation values for each gelatine concentration. Standard deviations were associated with the intrinsic variability of using different samples and rheometry technique. Results at frequencies higher than 4.5 Hz showed instabilities and incoherent values for all the tested samples. At higher frequencies, inertial effects of the rheometer could have altered the measurements. This phenomenon is common in rheometry tests^37^. Overall, higher concentrations of gelatine provided higher values of both $G'$ and $G''$. Fluctuating values were observed at frequencies below 0.7 Hz. Nevertheless, this phenomenon was not relevant to the aim of the rheometry tests. One plausible reason was the loss of sensitivity of the rheometer at very low frequencies.

The ratio between the absolute values of $G^*$of the stiffer inclusions over the background gelatine batches, hereinafter named modulus contrast ratio $\left| G^*\right| _{ratio}$, was calculated. Averaged values of this contrast ratio were 1.2, 1.7 and 3.8 for the gelatine concentrations of 12, 14 and 16%, which confirms that higher gelatine concentration produces higher stiffness.

### HSC tests results

**Figure 9:**
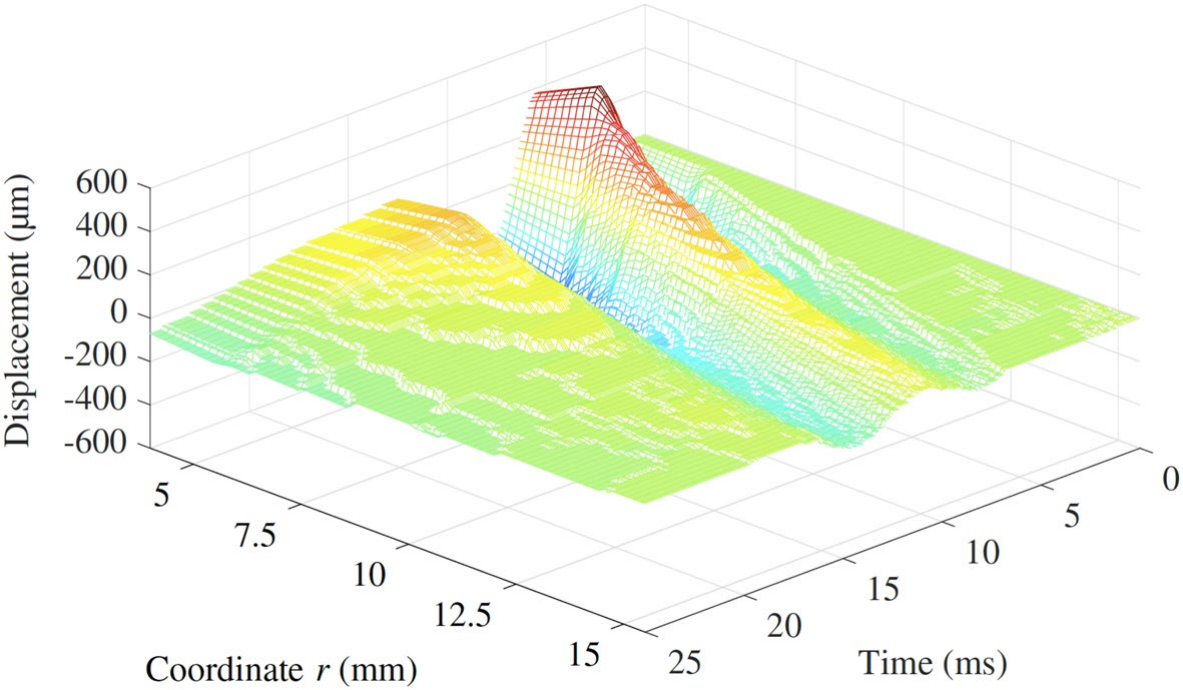
Time-space representation of the particle displacement due to the shear wave propagation for the illustrative case in plane A. The propagation of the shear wave was recorded for 25 ms. The *r* coordinate ranged from 4 to 15.5 mm, with *r* = 0 corresponding to the centre of the disk emitter, and the inclusion front face at approximately *r* = 15 mm. Although the driving signal was a single sinusoidal cycle, the displacement of the emitter (visible at *r* = 4–5.5 mm) showed a noticeable extension due to the inertia of the phantom.

HSC tests were carried out on the three fabricated phantoms. The camera captured the displacement of the basalt particles located in the planes A and B due the propagation of the shear wave generated. Results were time-space representations of the displacement generated by the shear wave propagation along the line of analysis. An example case was selected to illustrate the results of the HSC tests in the time-space domain (Fig. 9). This case consisted of the phantom with the inclusion with the highest modulus contrast ratio, 3.8, and centre frequency of the wave propagated at 150 Hz. This particular scenario is referred to as the illustrative case throughout the rest of the article.

### Characterisation of the KVFD parameters of the phantoms

**Figure 10:**
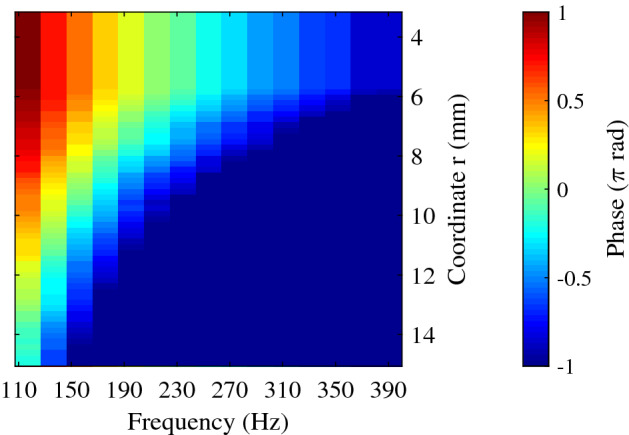
Example of a phase spectrum calculated over the line of analysis for the illustrative case in the plane B.

From the rheometry tests, each phase velocity was calculated using Eqs. (8), (9) and (10). In the case of the HSC tests, phase velocity was calculated using Eq. (7) after obtaining the phase spectra along the line of analysis (see example for the illustrative case plane B in Fig. 10). Although the single pulses of the driving signal had frequencies ranging from 100 to 1000 Hz, the final transmitted waves contained a broad frequency spectrum with lower centre frequency, due to the viscoelastic response of the medium and the insufficient torque of the emitter to overcome the inertia.

**Figure 11:**
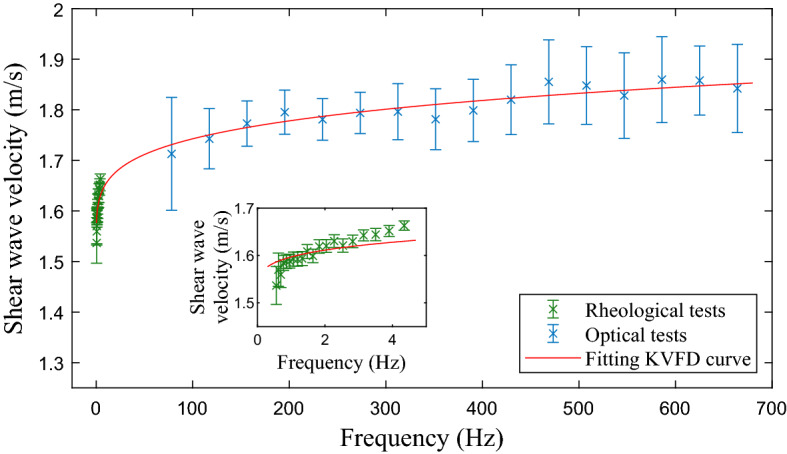
Shear wave velocity dispersion curve over frequency for the background material of the phantoms by combining results from the rheometry tests (in green) and from the HSC tests (in blue). The red curve is the result of fitting the data by the theoretical KVFD expression for shear wave velocity. Results are shown in terms of mean and standard deviation values after combining results from the three phantoms.

The KVFD viscoelastic properties of the phantoms, needed for building the wave propagation model, were obtained from the velocity dispersion curve of the phantom material. The velocity dispersion curve (Fig. 11) resulted by fitting the combination of results from the rheometry tests and the particle displacement reconstructed from the HSC recordings to the theoretical velocity dispersion expression (replacing Eqs. (9) and (10) into Eq. (8)). The KVFD parameters obtained were $\mu$ = 2.61 kPa, $\eta$ = 205.2 Pa s$^\alpha$ and $\alpha$ = 0.2117, with goodness-of-fit R$^2$ = 0.9610. As can be seen in the magnified view insert in Fig. 11, the fitted KVFD curve slightly misses the trend of the rheological data. However, the overall fit remained satisfactory, as the range of frequency of the rheological tests was much narrower than the total frequency range after adding the data from the optical tests.

### Validation of the wave propagation model

The wave propagation model was used to replicate the observations from the HSC tests. The KVFD parameters derived above were set into the wave propagation model (summarised in Table 4). The modulus contrast ratios measured in the rheometry tests between gelatine mixtures was used to derive the KVFD parameters for the inclusions (using Eqs. 9 and 10). The parameter $\alpha$ was kept constant for all the inclusions as in previous articles for the contrast between cancerous and healthy tissue^4,38^.

**Table 4:** KVFD parameters used for the wave propagation model simulations.

Gelatine mixture	$\left| G^*\right| _{ratio}$	KVFD parameters		
		$\mu$ (kPa)	$\eta$ (Pa s$^\alpha$)	$\alpha$
Background	1.0	2.61	205.2	0.21
Inclusion 1	1.2	3.13	246.2	0.21
Inclusion 2	1.7	4.44	348.8	0.21
Inclusion 3	3.8	9.92	779.8	0.21

**Figure 12:**
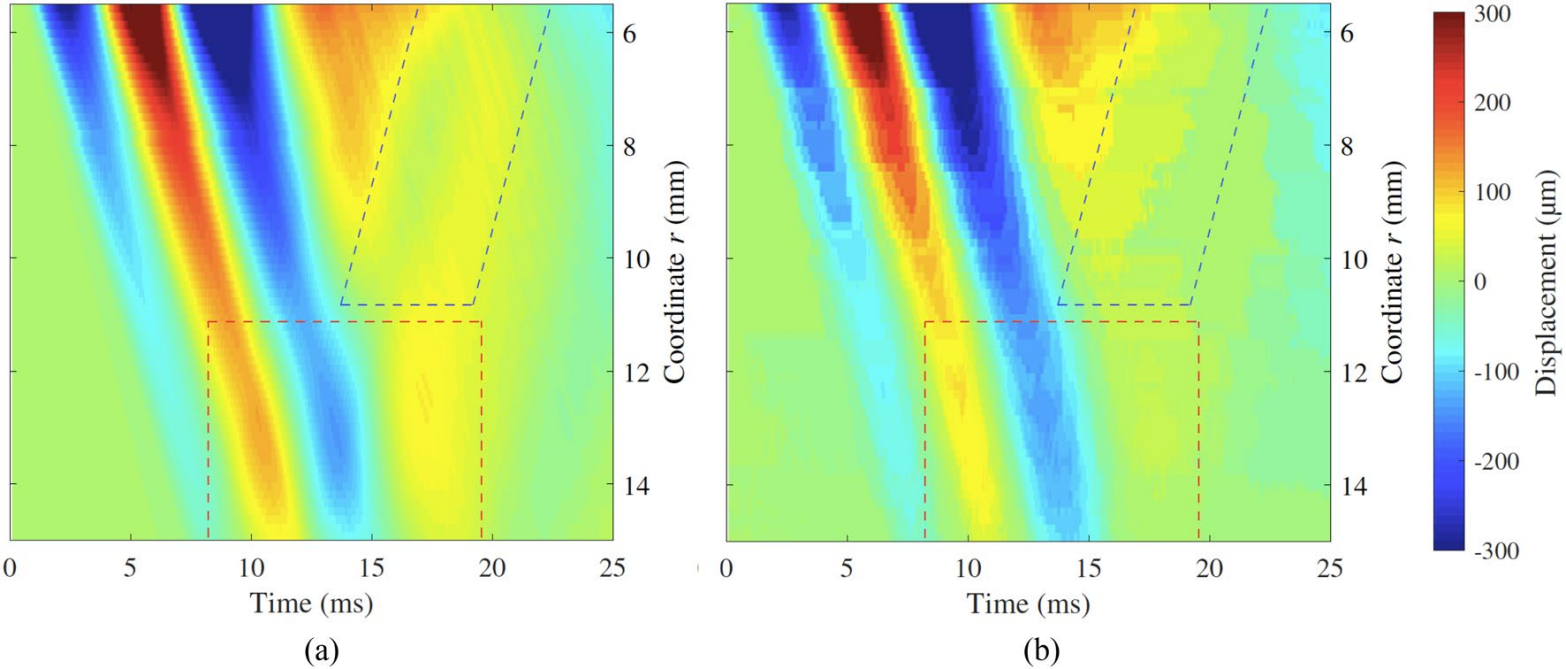
Time-space representation of the wave propagation for the illustrative case, plane A, i.e. with an inclusion. The colour maps represent the particle displacement $u_\theta$ for (**a**) the model simulation and (**b**) the HSC test. The top side of the figures (*r* = 5.5 mm) coincides with the edge of the disk emitter, while the bottom side coincides with the front face of the stiff inclusion (*r* = 15 mm). The perturbation due to the interaction between the incident wave and its reflection against the stiff inclusion is observable within the regions contoured by dashed red line. The reflection continued propagating back to the emitter location and is noticeable within the regions contoured by dashed blue line.

**Figure 13:**
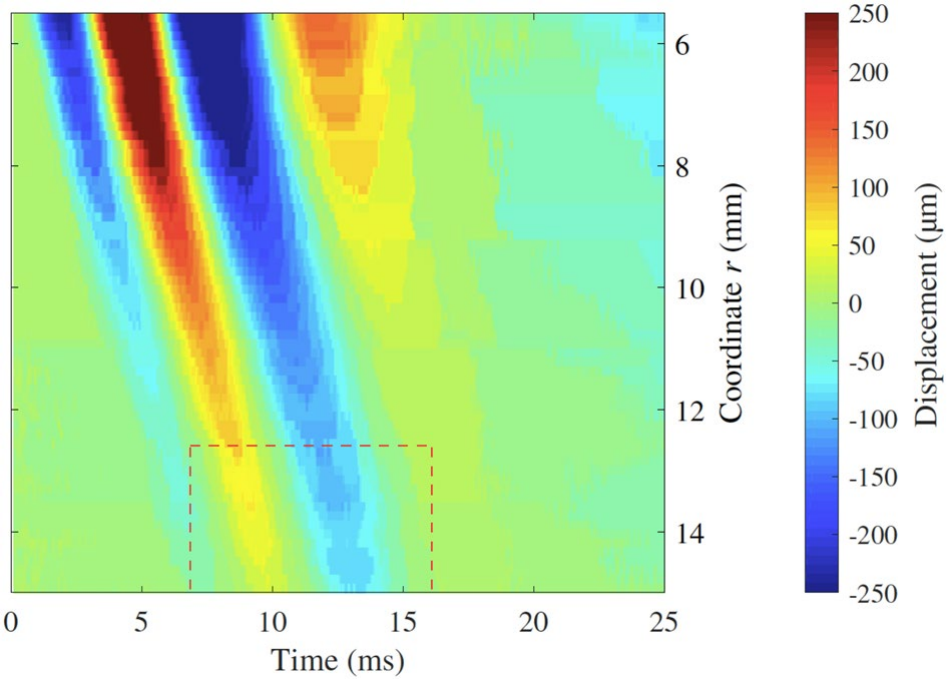
Time-space representation of the particle displacement $u_\theta$ from a HSC test in phantom 1. Driving signal at 300 Hz. The top side of the figure coincides with the edge of the disk emitter, while the bottom side coincides with the front face of the stiff inclusion.

A simulation example for the illustrative case in plane A is shown in Fig. 12a. The result from the HSC test for the illustrative example is shown in Fig. 12b. Both the model simulation and the HSC result yielded similar features in terms of time-space propagation. A perturbation generated by the reflection was noticeable in both simulation and HSC results, as changes in the apparent wave group velocity. Apparent wave group velocity is here understood as the slope of the line that contained the wave peaks of the time-space representation (see regions within the dashed red boxes in Fig. 12). Nevertheless, there were some slight differences in terms of amplitude of the reflected energy going back to the emitter (see regions within the dashed blue polygons in Fig. 12). The perturbation in the proximity of the inclusion was consistently observed in all the tests performed in phantom 3, whilst in the other two, the perturbation was less noticeable. Phantom 3 contained the inclusion with the highest modulus contrast ratio, hence the highest change in shear acoustic impedance, which provided the highest reflection coefficient among the three phantoms. An example of this is shown in Fig. 13, which corresponds to the results from the HSC test in phantom 1. A weaker perturbation was observed in the proximity of the inclusion (see region within the dashed red box in Fig. 13).

**Figure 14:**
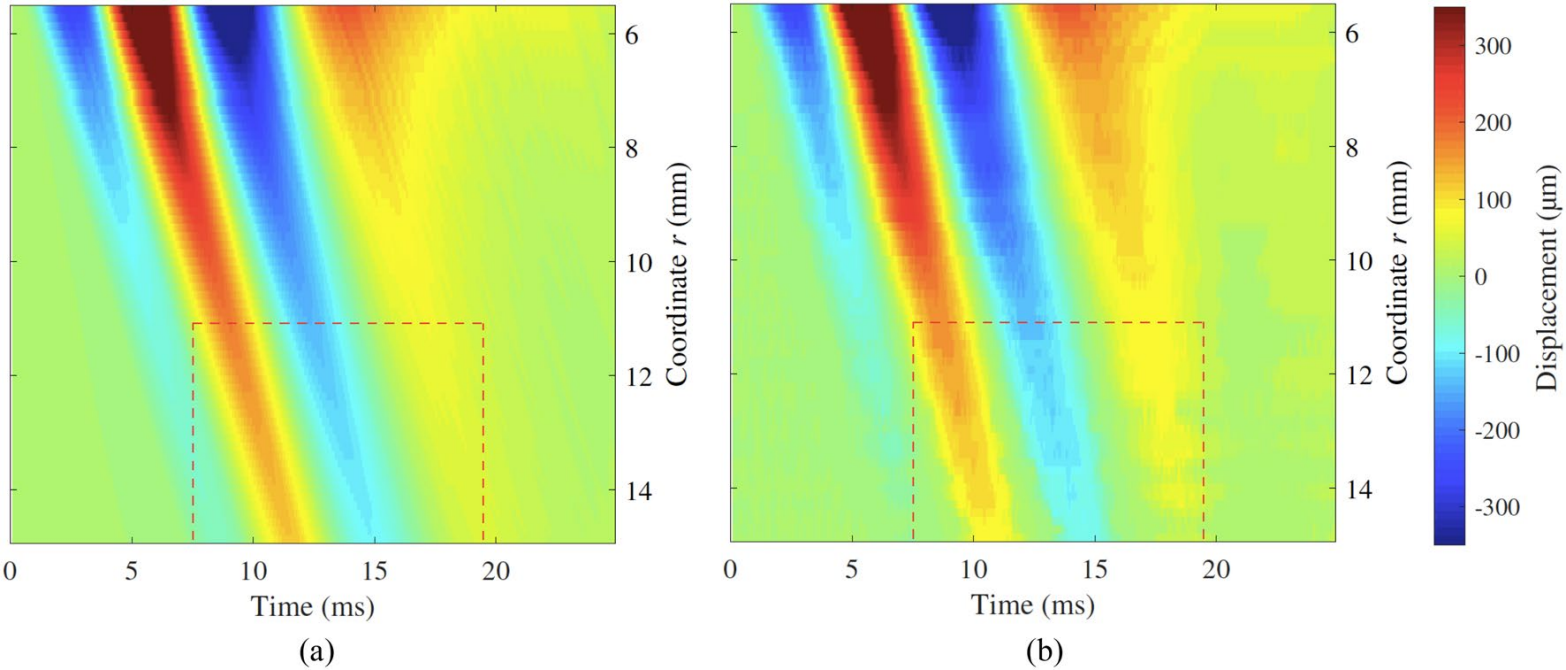
Time-space representation of the wave propagation in the illustrative case for plane B, i.e., with no inclusion. The colour maps represent the particle displacement $u_\theta$ for (**a**) the model simulation and (**b**) the HSC test.

Figure 14 shows a visual comparison between model simulation and reconstruction from the HSC test for the illustrative case but in the plane B, i.e., with no inclusion. The perturbation visible in the previous example (Fig. 12) is not present because in the plane B no inclusion was present. This was consistently observed in all phantoms for plane B, thus confirming that the perturbations observed were due to the presence of stiff inclusion.

Overall, the model simulations replicated well the results from the HSC tests, with some differences in the amplitude of the reflected wave in the proximity of the location of the emitter. The correlation coefficient $r_{Pearson}$ was calculated for each pair of simulation and HSC test, with values between 0.9671 and 0.9894, mean value of 0.9785 and standard deviation of 0.0068. These values, close to 1, indicated that there was a high similarity between model simulations and HSC tests in terms of time-space propagation features.

**Figure 15:**
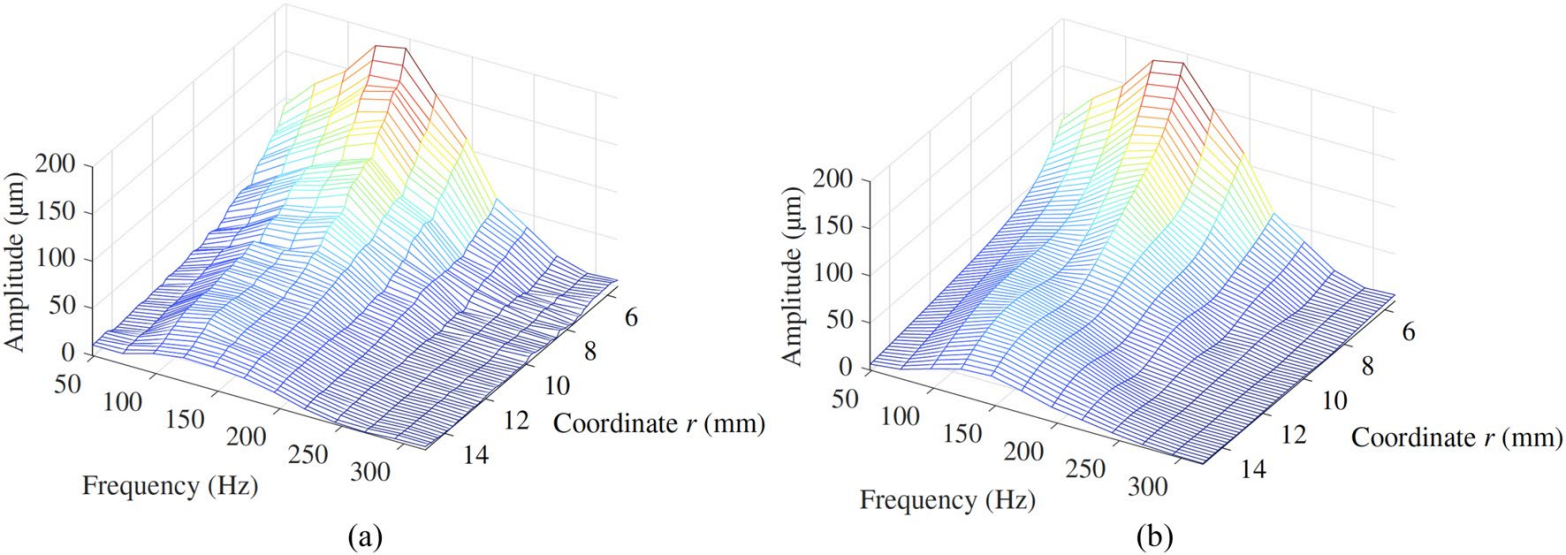
Amplitude spectrum from (**a**) the HSC test and (**b**) the model simulation, for the illustrative case in plane A.

The second validation approach was based on a quantitative comparison of the amplitude spectra of the model simulations and the particle displacement reconstructed from the HSC tests. For this analysis, only the forward propagation was considered, i.e., for the plane A cases, the propagation before reaching the inclusion was analysed. An example of amplitude spectrum from the HSC test in the illustrative case and the corresponding model simulation are shown in Fig. 15a and b, respectively. The axis *r* represents the distance of propagation along the line of analysis. As in the time-space graphs from Figs. 12, 13 and 14, the edge of the disk emitter coincides with *r* = 5.5 mm and the interface between background and inclusion is located at *r* = 15 mm. The normalised RMSE was calculated as a metric of the difference between the amplitude spectra from the model simulations and the experimental cases. Overall, the values of the normalised RMSE were between 2.36 and 5.77%, with a mean value of 3.76% and standard deviation of 1.20%. These errors were considered relatively low, thus concluding that the amplitude of the wave and its attenuation, before reflection, were correctly replicated by the wave propagation model.

## Discussion

This article describes the experimental validation of the wave propagation model for a novel transluminal elastography procedure presented in a previous article^4^. The validation approach was based on the comparison between model simulations and HSC observations of the particle vibration field generated by shear waves in soft tissue-like phantoms. Previous works that have used HSC-based techniques for measuring shear waves only captured the vibration of single particles^19–21^. The HSC-based method described here permitted the observation of hundreds of particles contained in a plane, which provide more information for reconstructing the particle vibration field.

In the previous article, prostate cancer detection was chosen to illustrate the model in silico and to serve as an example application for the novel transluminal procedure. Following the same rationale, the phantom recipe was adjusted so the KVFD viscoelastic properties of the phantoms matched those of prostatic tissue. Three phantoms were built containing an inclusion with different stiffness. Characterisation of the KVFD viscoelastic properties of the different phantom materials was needed for the model simulations. The KVFD properties were derived from the shear wave velocity dispersion curve built by combining results from rheometry tests performed with frequencies of up to 5 Hz, and the HSC tests with frequencies ranging from 80 to almost 700 Hz.

Only shear wave velocities at frequency below 5 Hz were obtained from the rheometry tests, as values at higher frequencies were dominated by inertial instability. This type of instability has commonly been reported in rheometers where the rotation sensor and the driving motor are mounted on the same shaft^37,39^, such as the rheometer used in this work. Rheometers with separated driver motor and sensor will be sought for future work, as this type has been proven to have a better performance against inertial instabilities^40^. The rheometry results also yielded the shear modulus contrast ratio between the background gelatine and the inclusions. The ratios obtained went from 1.2 to 3.8, which agree with those found in prostate cancer^41,42^.

HSC tests were carried out to capture the displacement field of particles inside the phantoms due to the propagation of shear waves according the transluminal procedure. Each phantom contained two planes with particles, plane A and B. Every plane A contained a stiff inclusion while plane B did not. A custom particle tracking algorithm reconstructed the particle displacement field from the camera recordings. HSC results were expressed as time-space graphs of the particle displacement along the lines of analysis. Phase velocities between 1.6 and 1.95 m/s were derived from the HSC results from the calculation of the phase shifts along the lines of analysis. These results were in agreement with values found for prostatic tissue in the literature^33^.

Comparison between model simulations and HSC observations was carried out by two different metrics. The first one, in the time domain, consisted of the correlation analysis between time-space graphs from model simulations and HSC tests. The obtained $r_{Pearson}$ coefficient averaged for all the tests was 0.9785, which indicates that the model and experimental observations were significantly similar in terms of time-space propagation features. HSC tests in planes A captured perturbations in the proximity of the inclusion’s front face (see dashed red boxes in Fig. 12), as a change in the apparent velocity of the wave. HSC observations in planes B showed no perturbation at all, which confirmed that the perturbations were due to the presence of the inclusions. Furthermore, these perturbations were more noticeable for model simulations and HSC tests in phantom 3, which had the highest modulus contrast ratio, in comparison with the other two phantoms where the perturbations were weaker (see example for phantom 1 in Fig. 13). In the model simulations there was energy reflected back to the emitter’s location visible after 15 ms of propagation (see the dashed blue box in Fig. 12a). These reflections were hardly visible in the HSC results. Furthermore, the perturbation nearby the inclusions in the HSC tests had a slightly different change in apparent velocity. It is believed that the geometrical differences between the model and the phantoms were responsible for these mismatches. The fabricated inclusions were straight cylinders, whereas the axisymmetric wave propagation model considered toroidal inclusions. Toroidal inclusions in the model create a concave curved interface that produces reflections converging onto the emitter. This results in less attenuation in contrast with straight interfaces that produce reflections that diffract, hence with higher attenuation. It was believed that in the experimental cases, the attenuation due to the added diffraction reduced the amplitude of the reflections to a level below the sensitivity of the HSC tests. In addition, casting the stiff inclusions could have generated a region of smooth transition between inclusion and background, thus creating a diffusive interface that damps the reflection.

A second comparison, this time in the frequency domain, was carried out to assess the ability of the model to replicate the amplitude and attenuation of the experimental tests. The data from planes A only considered the time-space propagation before encountering the inclusions. Quantitative comparison using the RMSE between amplitude spectra from both the model simulations and the HSC tests was carried out. Results averaged from all the tests showed a mean RMSE value of 3.76% and standard validation of 1.20%, which can be considered relatively low.

Overall, the significant similarities obtained between the experimental results and the wave propagation model, specifically the propagation features of the forward and reflected waves, as well as the amplitude attenuation of the forward wave, were considered sufficient to validate the wave propagation model developed for the transluminal elastography procedure. The HSC technique described here can be a useful tool for observing shear wave propagation in translucent phantoms at frequencies up to 700 Hz. Finally, these results, together with the observations from a previous article^3^, present the preliminary proof-of-concept for the novel transluminal elastography procedure shown in Gomez et al.^4^.

## Data Availability

The data sets used and/or analysed during the current study are available from the corresponding author on reasonable request.
